# Postmenopausal women with osteopenia and a healed wrist fracture have reduced physical function and quality of life compared to a matched, healthy control group with no fracture

**DOI:** 10.1186/1472-6874-14-92

**Published:** 2014-08-03

**Authors:** Kari Anne Hakestad, Lars Nordsletten, Monica Klungland Torstveit, May Arna Risberg

**Affiliations:** 1 Department of Orthopaedic Surgery, Norwegian Research Center for Active Rehabilitation (NAR), Oslo University Hospital, Trondheimsveien 235, 0514 Oslo, Norway; 2 University of Oslo, Oslo, Norway; 3 Department of Sport Medicine, Norwegian School of Sport Sciences, Oslo, Norway; 4 Faculty of Health and Sport Sciences, University of Agder, Oslo, Norway

**Keywords:** Osteopenia, Quadriceps strength, Dynamic balance, Postmenopausal women, Wrist fracture

## Abstract

**Background:**

Fractures lead to reduced physical function and quality of life (QOL), but little is known about postmenopausal women with osteopenia and a healed wrist fracture. The purpose was to evaluate physical function in terms of quadriceps strength, dynamic balance, physical capacity and QOL in postmenopausal women with osteopenia and a healed wrist fracture compared to a matched, healthy control group with no previous fracture.

**Methods:**

Eighteen postmenopausal women with osteopenia (patients) (mean age 59.1 years, range 54 – 65) and a healed wrist fracture were matched to 18 healthy control subjects on age (mean age 58.5 years, range 51 – 65), height, weight and body mass index (BMI). We measured quadriceps strength at 60°/sec and at 180°/sec with Biodex 6000, dynamic balance with the Four Square Step Test (FSST), physical capacity with the six-minute walk test (6MWT) followed by the Borg’s scale (BS), and QOL with the Short Form 36 (SF-36), bone mineral density (BMD) with dual x-ray absorptiometry (DXA) and physical activity level with the Physical Activity Scale for the Elderly.

**Results:**

The patients had 17.6% lower quadriceps strength at 60°/sec (p = 0.025) at left limb and 18.5% at 180°/sec (p = 0.016) at right limb, and 21% lower at 180°/sec (p = 0.010) at left limb compared to the controls. Impaired performance for the patients was found with 2.4 seconds (p = 0.002) on the FSST, 74 metres (p < 0.001) on the 6MWT, and 1.4 points (p = 0.003) on the BS compared to the controls. The patients scored lower on the sub-scales on the SF-36 role limitations-physical (p = 0.014), bodily pain (p = 0.025) and vitality (p = 0.015) compared to the controls.

**Conclusions:**

The patients with osteopenia and a healed wrist fracture scored significantly lower on quadriceps strength, dynamic balance, physical capacity and QOL compared to the matched controls. Greater focus should be put on this patient group in terms of rehabilitation and early prevention of subsequent fractures.

## Background

Fractures in the elderly lead to reduced physical function and quality of life (QOL), as well as increased mortality and higher healthcare costs [1-3]. As a consequence of the increasing number of elderly people in the population and of the gradual reduction of bone mineral density (BMD), muscle mass and muscle strength and the loss of hormone production, the number of osteoporotic fractures will increase in the years to come [4]. This may therefore become a major public health problem [5].

Elderly patients have an increased tendency to fall due to reduced muscular strength and impaired balance [6]. Most fractures result from a fall [7]. In one year, approximately 35-40% of those over 65 years of age fall at least once, and about half of these fall twice or more [8]. It has also been proven that a previous wrist fracture is a risk factor for future hip or vertebral fractures with a relative risk (RR) of 1.9 and 4.4 respectively [9]. We know that among those who already have sustained a fracture, 50% will experience a new fracture within a ten-year period [10].

Several studies have found reduced muscle strength, impaired balance, reduced physical capacity, increased fear of falling (FOF) and reduced QOL among patients with low BMD (osteopenia or osteoporosis) [3,11-16]. However, the majority of the existing studies have included women with hip or vertebral fractures [3,11-16]. Since a hip or vertebral fracture may directly influence the above functions, a wrist fracture could be a good model to evaluate the effect of the disease behind the fracture. Some studies investigating women with osteopenia and a healed wrist fracture have reported functional impairment and lower QOL compared to a healthy control group [17,18]. In contrast, other studies have not found any differences between corresponding groups [19,20].

The aim of the present study was therefore to evaluate physical function in terms of quadriceps strength, dynamic balance, physical capacity, physical activity level and QOL in postmenopausal women with osteopenia and a healed wrist fracture compared to a matched, healthy control group with no previous fracture.

## Methods

### Design

A cross-sectional study with a matched control group was conducted in the period 2007–2010.

### Matching procedure

**Figure 1:**
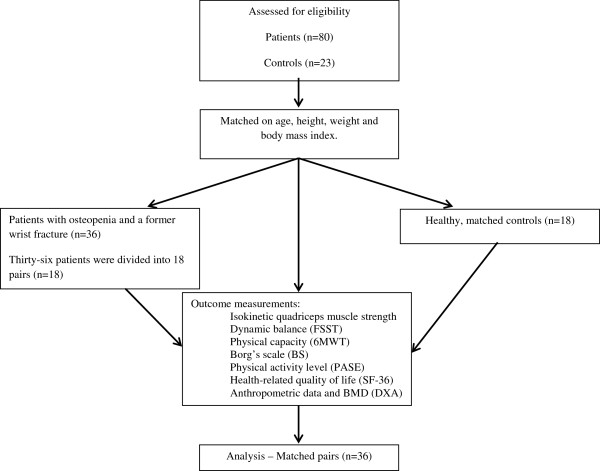
Flow-chart of the inclusion of the subjects.

The matching procedure was as follows: we recruited the healthy controls from the local area by healthcare professionals and through patients’ friends. They were matched on age (±5 years), height, weight and body mass index (BMI) among the 80 included subjects in the on-going randomized, controlled, single-blinded study (RCT) (reference number http://www.clinicaltrials.gov NCT01357278) carried out at Oslo University Hospital, Norway who were most similar to the controls on the selected criteria. In total 36 patients in the RCT matched the 18 selected controls. To have a 1:1 relationship we divided the 36 into 18 pairs, and the demographic characteristics of both members of the pair were then averaged to derive a single value for the pair to which controls were then matched.The main aim of the RCT was to evaluate the effect of an active rehabilitation programme using weight vests on risk factors for falling (quadriceps strength, balance) and quality of life in women with osteopenia and a healed wrist fracture. The intervention consisted of a six-month active rehabilitation programme with a one-year follow-up (Figure 1).

### Patients

The inclusion and exclusion criteria were similar to those used in the RCT. Thirty-six postmenopausal women above 50 years of age with a wrist fracture no older than two years, healed at inclusion (no plaster cast), with osteopenia (t-score < 1.5) [1] and domiciled in the Oslo region were recruited at baseline from the on-going RCT. The patients were excluded if they had a previous hip or vertebral fracture, a history of more than three osteoporotic fractures in different parts of the body, problems/illness indicating that active rehabilitation was not suitable, were physically active (moderate/hard intensity) more than four hours per week, or were unable to understand written and spoken Norwegian.

### Control group

Eighteen healthy postmenopausal women above 50 years of age were recruited from the local area by colleagues and through patients’ friends. The controls were excluded if they had previous fractures, were diagnosed with osteoporosis, were physically active (moderate/hard intensity) for more than four hours per week, or were unable to understand written and spoken Norwegian.

Ethical approval was obtained from the Regional Committee for Medical Research Ethics for South-Eastern Norway. All participants who met the inclusion criteria received oral and written information about the study, and signed informed consent. The data collection was carried out in accordance with the Declaration of Helsinki. The study has adhered to the STROBE guidelines.

### Measurements

Isokinetic quadriceps strength was the main outcome and was examined with a Biodex 6000 isokinetic dynamometer (Biodex 3 System Pro, USA). Peak torque in Newton metres (Nm) at 60° and total work in Joule (J) at 180° per second were measured. Our research group has found isokinetic muscle strength test to have high inter- and intrarater reliability (ICC 0.88-0.95) in postmenopausal women with osteopenia [21].

Dynamic balance was evaluated with the Four Square Step Test (FSST) [22]. Two FSSTs were completed using the best score of the two trials. The FSST has shown high reliability (ICC = 0.99), and validity with a sensitivity of 85% and a specificity of 88% to 100% [22].

Physical capacity was examined with the six-minute walk test (6MWT) [23]. The 6MWT has been validated for measuring the functional capacity of elderly people [23]. Immediately following the 6MWT, the participants were asked how exhausting they experienced the 6MWT using Borg’s scale (BS) ranging from 6–20, where 6 indicates “very easy” and 20 indicates “very exhausting” [24].

QOL was measured by means of the Short Form 36 (SF-36) [25]. The SF-36 is divided into eight sub-scales (physical function, role limitations-physical, bodily pain, general health, vitality, social function, role limitations-emotional and mental health). The instrument is scored to a 0–100 scale for each sub-scale, the higher the score, the better the health status [25]. The SF-36 is shown to have high reliability and validity [26].

BMD was measured by means of dual x-ray absorptiometry (DXA, GE Lunar scan Prodigy, enCORE version 11.2). The method is exact and reliable [1,27], and the scanned areas were hip, femur neck and trochanter, lumbar spine and total body. Anthropometric data such as height and weight were measured by means of weight- and height scales and absolute and percentage fat, plus fat-free mass were measured with DXA.

Physical activity level was registered with the self-reported level of Physical Activity Scale for the Elderly (PASE) [28]. We used the modified Norwegian version [29]. The score range from 0–315, where higher scores reflect a higher activity level [29].

The measurements were conducted at two visits. At the first visit the anthropometric data and BMD were registered by using DXA for meeting the inclusion criteria. If the criteria were met, the participants first filled in questionnaires regarding socio-economic factors, PASE and SF-36. They then performed the FSST and the 6MWT followed by the BS. Quadriceps strength was examined at the second visit.

### Data analysis

The sample size was based on the formula for case–control studies with continuous variables [30]. The main outcome measurement was isokinetic quadriceps strength peak torque values at 60°/sec in Nm with a mean of 151.4 (±28.7) for subjects with no fracture and a mean of 126.4 (±42.3) Nm for patients with osteopenia and a healed wrist fracture (unpublished observations by Hakestad, Kari Anne; Risberg, May Arna). With a statistical significance of p < 0.05 and a power of 90%, 17 subjects were needed for inclusion in each group. We took the option of taking a 2:1 ratio (2 cases: 1 control) between cases and controls to increase the power of the study. The analyses were carried out with the SPSS version 19.0. The paired samples Student’s *t*-test was used to evaluate differences between the groups for normally distributed data. The results are presented as mean, mean difference, and 95% confidence interval (CI) and p-values.

## Results

There were no significant differences between the patients and the matched, healthy controls with regard to age, height, weight, BMI, age of menopause, years since postmenopause, hand dominance, body fat, lean mass, PASE and education. An additional table file shows this in more detail (see Additional file 1). The mean years since the time of the wrist fractures to inclusion were 1.3 years (0.6). Ten of the patients had the fracture in the right hand, eight in the left hand (see Additional file 1). The patients had 16% lower BMD in the lumbar spine (p < 0.001), 15% lower BMD in the total hip (p < 0.001), 14.5% lower BMD in the femur neck (p < 0.001) and 18% lower BMD in the femur trochanter (p < 0.001) compared to the controls (see Additional file 1).

The patients had 17.6% lower quadriceps strength at 60°/sec (p = 0.025) at left limb (Table 1), but no significant difference was obtained in the right limb (Table 1), and at 180°/sec the right and left limb were 18.5% (p = 0.016) and 21% (p = 0.010) lower respectively compared to the controls (Table 1).

**Table 1:** Quadriceps strength in patients with osteopenia and a healed wrist fracture (n = 18) and matched controls (n = 18)

			**Mean difference**	**P Value**
	**Patients**	**Controls**	**(95% CI)**	
Right peak torque 60° (Nm)	102.4	113.2	-10.8 (-26.9 to 5.4)	0.178
Left peak torque 60° (Nm)	96.4	115.0	-18.6 (-34.6 to -2.6)	0.025
Right total work 180° (J)	1276.8	1536.7	-259.9 (-464.9 to -54.9)	0.016
Left total work 180° (J)	1194.8	1480.4	-285.6 (-492.7 to -78.5)	0.010

Data are presented as mean, *CI* Confidence Interval, *Nm* Newton metres, *J* Joule.

For the dynamic balance, the patients had impaired performance of 2.4 seconds (p = 0.002) compared to the controls (Table 2). The walking distance during the 6MWT was 74 metres (p < 0.001) shorter for the patients compared to the controls (Table 2). Furthermore, the patients reported 1.4 points (p = 0.003) higher exhaustion levels on BS after completing the 6MWT compared to the controls (Table 2).

**Table 2:** Dynamic balance, Six-minute walk test, and Borg scale in patients with osteopenia and a healed wrist fracture (n = 18) and matched controls (n = 18)

			**Mean Difference**	**P Value**
	**Patients**	**Controls**	**(95% CI)**	
FSST (sec)	9.4	7.0	2.4 (1.0 to 3.7)	0.002
6 MWT (m)	615.7	690.0	-74.3 (-105.4 to -43.0)	<0.001
Borg scale	10.2	8.8	1.4 (0.5 to 2.3)	0.003

Data are presented as mean, *CI* Confidence Interval.

*FSST* Four Square Step Test, *6MWT* Six-minute walk test.

QOL was lower among the patients compared to the controls in the sub-scales role limitations-physical (10.2 points; p = 0.014), bodily pain (11.9 points; p = 0.025) and vitality (16.9 points; p = 0.015) respectively (Table 3).

**Table 3:** Subscales of the SF-36 in patients with osteopenia and a healed wrist fracture (n = 18) and matched controls (n = 18)

			**Mean difference**	**P value**
	**Patients**	**Controls**	**(95% CI)**	
Physical functioning	90.0	95.3	-5.3 (-10.7 to 0.1)	0.056
Role limitations-physical	86.6	96.8	-10.2 (-18.1 to -2.3)	0.014
Bodily pain	76.5	88.4	-11.9 (-22.2 to -1.6)	0.025
General health perceptions	77.0	79.7	-2.7 (-12.9 to 7.6)	0.596
Vitality	58.1	75.0	-16.9 (-29.9 to -3.7)	0.015
Social functioning	89.9	96.5	-6.6 (-14.6 to 1.4)	0.100
Role limitations-emotional	91.4	97.7	-6.3 (-12.6 to 0.7)	0.053
Mental health	86.4	88.9	-2.5 (-9.9 to 4.8)	0.483

Data are presented as mean, *CI* Confidence Interval.

*SF-36* Short Form 36.

## Discussion

We found significantly lower quadriceps strength in women with osteopenia and a healed wrist fracture compared to healthy, matched controls. Furthermore, the patients had impaired physical performance measured as dynamic balance (FSST), walking distance (6MWT) and exhaustion level (BS), as well as in three dimensions of QOL (SF-36) compared to the controls.

Studies have investigated the quadriceps strength in women with hip or vertebral fractures and have found that quadriceps strength was 18% lower compared to a control group or the non-fractured limb [13,14]. To our knowledge, no studies have examined the quadriceps strength in postmenopausal women with osteopenia and a healed wrist fracture compared to a matched, healthy control group. When comparing the data from the above studies [13,14], we found similar results for patients with a wrist fracture with 18-21% differences in quadriceps strength between our patients and controls indicating a clinically important difference. This was confirmed by our research group Eitzen et al. [21] who found that a minimal clinically important difference (MCID) in knee extension was suggested to be between 15% and 20%. Furthermore, it may not be surprising that our patients had between 18-21% lower quadriceps strength than the controls. Along with age, weight and estrogen levels, low muscle strength have been shown to be one of the most important factors influencing BMD [31]. A recent systematic review found that there was a correlation between low BMD in the hip region and poor muscle strength in the lower limb [32]. One of the reasons could be the site-specific effect that is generated by muscle contraction on bone remodelling [33]. Genetic factors may also play a major role [31]. It has been shown that there are components of BMD and muscle strength which are significantly controlled by genes [31]. The lower quadriceps strength among the patients compared to the controls may explain the impaired dynamic balance among our patients. Earlier studies have shown that weak balance is associated with reduced quadriceps strength [34]. Stiffness of the joints can increase the risk of falls due to changed proprioception and reduced muscle strength [34,35], and a correlation between poor muscle strength in both the upper and lower extremities has been reported [34].

Our patients spent significantly more time performing the FSST than the controls. This is in accordance with a study conducted by Ringsberg et al. [19] who found impaired dynamic balance in women with previous wrist fracture compared to a non-fracture control group. They suggested that the impaired balance could be an expression of fear of falling (FOF) [19]. Approximately 25-60% of the elderly population experience FOF [36], which can lead to reduced self-esteem, both mentally and physically [36]. Moreover, FOF can also lead to physical inactivity, which can result in muscle weakness, falls and subsequent fractures [36].

Our patients had 74 metres shorter 6MWT compared to the controls. To our knowledge, no studies have reported MCID in the 6MWT in patients with low BMD. However, Perera et al. [37] reported that 50 metres is considered to be an MCID in the 6MWT in the elderly. Hicks et al. [38] concluded that a decline in muscle strength is considered an important factor for the walking speed. Furthermore, the patients experienced significantly more exhaustion on BS when performing the 6MWT compared to controls. This could indicate that the patients really performed up towards their maximum and that the controls could potentially have walked even faster.

Nordvall et al. [18] examined the QOL in postmenopausal women with low BMD and wrist fracture compared to a healthy control group and found significant differences for the physical role and bodily pain dimensions of the SF-36. Hallberg et al. [11] reported that QOL using the SF-36 was reduced in the first six months after a wrist fracture among postmenopausal women, and was not normalized until two years after the fracture had occurred. Our patients scored significantly lower for the dimensions of physical role with 10.2 points, bodily pain with 11.9 points and vitality with 16.9 points compared to the controls. As far as we know, no MCID for SF-36 has been reported for women with low BMD, but Hays et al. [39] have reported that an improvement of 3 to 5 points should be considered clinically relevant. Another study has reported that patients with wrist fracture had experienced pain and decreased physical function during the first weeks after the fracture, and patients who had sustained a wrist fracture had lower QOL, specifically for the dimensions of physical function and role-physical in SF-36 [40]. Furthermore, a negative correlation between osteoporotic fractures and QOL has been reported [11]. Even though patients with a healed wrist fracture seem to have the same level of QOL as they had prior to the fracture after two years [3], it is important to start early prevention of risk factors for falling because of the knowledge of the increased risk of future fractures [9].

### Strengths and limitations

Our study has some limitations. Firstly, the cross-sectional study design cannot identify causal relationships between the disease and the exposure [41]. To answer the research question better, the most appropriate design for studies where the aim is prospective is to use a cohort design (descriptive) for monitoring the natural history of a disease or injury or for evaluating a treatment [41]. Secondly, the power calculation was only based on the main outcome measure quadriceps strength, resulting in lack of statistical power for some of the other outcome measures. Thirdly, the controls were not randomly selected which may have resulted in selection bias. In addition, we were unable to adjust our finding by BMD and other covariates due to sample size constraints [42].

One of the major strengths of our study is being one of the first including a matched control group who had not sustained fractures, which enabled us to evaluate functional decline and QOL due to wrist fracture versus changes due to aging. Knowledge of physical function and QOL among postmenopausal women with osteopenia and a healed wrist fracture may help healthcare professionals to evaluate and develop rehabilitation programmes aimed at improving significant risk factors for falls, such as quadriceps strength and dynamic balance.

## Conclusion

Quadriceps strength, dynamic balance, physical capacity and QOL were significantly lower in postmenopausal women with osteopenia and a healed wrist fracture compared to a matched, healthy control group. Interventions to reduce fracture risk among patients with wrist fracture should be started as soon as possible to prevent subsequent fractures with their associated morbidity and increased mortality risk.

## Abbreviations

QOL: Quality of life; BMI: Body mass index; FSST: Four square step test; 6MWT: Six-minute walk test; BS: Borg’s scale; SF-36: Short form 36; BMD: Bone mineral density; DXA: Dual x-ray absorptiometry; RR: Relative risk; FOF: Fear of falling; RCT: Randomized, controlled trial; Nm: Newton metres; J: Joule; ICC: Intra-class correlation coefficient; PASE: Physical activity scale for the elderly; CI: Confidence interval; MCID: Minimal clinically important difference.

## Competing interests

The authors declare that they have no competing interests.

## Authors’ contributions

LN, MKT and MAR participated in the design of the study, contributed to drafting the article and read and approved the final manuscript. KAH carried out the patient inclusion, administered the questionnaires, performed all the physical examinations and conducted the statistical analysis. All the authors read and approved the final manuscript.

## Pre-publication history

The pre-publication history for this paper can be accessed here:

http://www.biomedcentral.com/1472-6874/14/92/prepub

## Supplementary Material

Additional file 1 Characteristics of patients with osteopenia and a healed wrist fracture (n = 18) and matched controls (n = 18). Click here for file

## References

[B1] Kanis JA Assessment of fracture risk and its application to screening for postmenopausal osteoporosis: synopsis of a WHO report. WHO Study Group Osteoporos Int 1994 4 368 381 10.1007/BF01622200 7696835

[B2] Osnes EK Lofthus CM Meyer HE Falch JA Nordsletten L Cappelen IF Kristiansen IS Consequences of hip fracture on activities of daily life and residential needs Osteoporos Int 2004 15 567 574 14730422 10.1007/s00198-003-1583-0

[B3] Pasco JA Sanders K Sanders KM Hoekstra F Hoekstra FM Henry M Henry MJ Nicholson G Nicholson GC Kotowicz M Kotowicz MA The human cost of fracture Osteoporos Int 2005 16 2046 2052 10.1007/s00198-005-1997-y 16228106

[B4] Ström O Borgström F Kanis JA Clompston J Cooper C McCloskey EV Jönsson B Osteoporosis: burden, health care provision and opportunities in the EU. A report prepared in collaboration with the International Osteoporosis Foundation (IOF) and the European of Pharmaceutical Industry Associations (EFPIA) Arch Osteoporos 2011 6 59 155 10.1007/s11657-011-0060-1 22886101

[B5] Johnell O Kanis JA An estimate of the worldwide prevalence and disability associated with osteoporotic fractures Osteoporos Int 2006 17 1726 1733 10.1007/s00198-006-0172-4 16983459

[B6] Lord SR Sherrington C Menz H Close J Falls in Older People: Risk Factors and Strategies for Prevention 2007 Cambridge: Cambridge University Press

[B7] Carter ND Kannus P Khan KM Exercise in the prevention of falls in older people: a systematic literature review examining the rationale and the evidence Sports Med 2001 31 427 438 10.2165/00007256-200131060-00003 11394562

[B8] Voermans NC Snijders AH Schoon Y Bloem BR Why old people fall (and how to stop them) Pract Neurol 2007 7 158 171 10.1136/jnnp.2007.120980 17515595

[B9] Klotzbuecher CM Ross PD Landsman PB Abbott TA III Berger M Patients with prior fractures have an increased risk of future fractures: a summary of the literature and statistical synthesis J Bone Miner Res 2000 15 721 739 10780864 10.1359/jbmr.2000.15.4.721

[B10] Kanis JA Johnell O Oden A Dawson A De Laet C Jonsson B Ten year probabilities of osteoporotic fractures according to BMD and diagnostic thresholds Osteoporos Int 2001 12 989 995 10.1007/s001980170006 11846333

[B11] Hallberg I Rosenqvist AM Kartous L Lofman O Wahlstrom O Toss G Health-related quality of life after osteoporotic fractures Osteoporos Int 2004 15 834 841 15045468 10.1007/s00198-004-1622-5

[B12] Hubscher M Vogt L Schmidt K Fink M Banzer W Perceived pain, fear of falling and physical function in women with osteoporosis Gait Posture 2010 32 383 385 10.1016/j.gaitpost.2010.06.018 20663672

[B13] Liu-Ambrose T Eng JJ Khan K Khan KM Carter N Carter ND McKay H McKay HA Older women with osteoporosis have increased postural sway and weaker quadriceps strength than counterparts with normal bone mass: overlooked determinants of fracture risk? J Gerontol A Biol Sci Med Sci 2003 58 M862 M866 14528046 10.1093/gerona/58.9.m862

[B14] Madsen OR Lauridsen UB Sorensen OH Quadriceps strength in women with a previous hip fracture: relationships to physical ability and bone mass Scand J Rehabil Med 2000 32 37 40 10.1080/003655000750045721 10782940

[B15] Magaziner J Fredman L Hawkes W Hebel JR Zimmerman S Orwig DL Wehren L Changes in functional status attributable to Hip fracture: a comparison of Hip fracture patients to community-dwelling aged Am J Epidemiol 2003 157 1023 1031 10.1093/aje/kwg081 12777366

[B16] Nguyen T Sambrook P Kelly P Jones G Freund LS Freund J Eisman J Prediction of osteoporotic fractures by postural instability and bone density BMJ 1993 307 1111 1115 10.1136/bmj.307.6912.1111 8251809 PMC1679116

[B17] Edwards BJ Song J Song J Dunlop DD Fink H Fink HA Cauley J Cauley JA Functional decline after incident wrist fractures--study of osteoporotic fractures: prospective cohort study BMJ 2010 341 c3324 10.1136/bmj.c3324 20616099 PMC2900548

[B18] Nordvall H Glanberg-Persson G Lysholm J Are distal radius fractures due to fragility or to falls? A consecutive case–control study of bone mineral density, tendency to fall, risk factors for osteoporosis, and health-related quality of life Acta Orthop Scand 2007 78 271 277 10.1080/17453670710013799 17464618

[B19] Ringsberg K Johnell O Obrant K Balance and speed of walking in women with Colles’ fractures Physiotherapy 1993 79 689 692 10.1016/S0031-9406(10)60002-8

[B20] Rohde G Haugeberg G Mengshoel AM Moum T Moum T Wahl AK No long-term impact of low-energy distal radius fracture on health-related quality of life and global quality of life: a case–control study BMC Musculoskelet Disord 2009 25 106 19706174 10.1186/1471-2474-10-106 PMC2751737

[B21] Eitzen I Hakestad KA Risberg MA Inter- and intrarater reliability of isokinetic thigh muscle strength tests in postmenopausal women with osteopenia Arch Phys Med Rehabil 2012 93 420 427 10.1016/j.apmr.2011.10.001 22265342

[B22] Dite W Temple VA A clinical test of stepping and change of direction to identify multiple falling older adults Arch Phys Med Rehabil 2002 83 1566 1571 10.1053/apmr.2002.35469 12422327

[B23] Enright PL The six-minute walk test Respir Care 2003 100 783 785 12890299

[B24] Borg GA Psychophysical bases of perceived exertion Med Sci Sports Exerc 1982 14 377 381 7154893

[B25] Ware JE Jr Sherbourne CD The MOS 36-item short-form health survey (SF-36). I. Conceptual framework and item selection Med Care 1992 30 473 483 10.1097/00005650-199206000-00002 1593914

[B26] McHorney CA Ware JE Jr Lu JF Sherbourne CD The MOS 36-item Short-Form Health Survey (SF-36): III. Tests of data quality, scaling assumptions, and reliability across diverse patient groups Med Care 1994 32 40 66 10.1097/00005650-199401000-00004 8277801

[B27] Blake GM FAU Fogelman I Fogelman I The role of DXA bone density scans in the diagnosis and treatment of osteoporosis Postgrad Med J 2007 83 509 517 10.1136/pgmj.2007.057505 17675543 PMC2600106

[B28] Washburn RA Smith KW Jette AM Janney CA The Physical Activity Scale for the Elderly (PASE): development and evaluation J Clin Epidemiol 1993 46 153 162 10.1016/0895-4356(93)90053-4 8437031

[B29] Loland N Reliability of the physical activity scale for the elderly (PASE) Eur J Sport Science 2002 2 1 12

[B30] Lubin JH Gail MH Ershow AG Sample size and power for case–control studies when exposures are continuous Stat Med 1988 7 363 376 10.1002/sim.4780070302 3358016

[B31] Bauer DC Browner WS Cauley JA Orwoll ES Scott JC Black DM Tao JL Cummings SR Factors associated with appendicular bone mass in older women. The study of osteoporotic fractures research group Ann Intern Med 1993 118 657 665 8460853 10.7326/0003-4819-118-9-199305010-00001

[B32] Shin H Panton LB Dutton G Dutton GR Ilich J Ilich JZ Relationship of physical performance with body composition and bone mineral density in individuals over 60 years of Age: a systematic review J Aging Res 2011 2011 191896 21318048 10.4061/2011/191896 PMC3034959

[B33] Blain H Vuillemin A Teissier A Hanesse B Guillemin F Jeandel C Influence of muscle strength and body weight and composition on regional bone mineral density in healthy women aged 60 years and over Gerontology 2001 47 207 212 10.1159/000052800 11408726

[B34] Horlings CG van Engelen B van Engelen BG Allum J Allum JH Bloem B Bloem BR A weak balance: the contribution of muscle weakness to postural instability and falls Nat Clin Pract Neurol 2008 4 504 515 10.1038/ncpneuro0886 18711425

[B35] Robstad B Frihagen F Nordsletten L The rate of hip osteoarthritis in patients with proximal femoral fractures versus hip contusion Osteoporos Int 2012 23 901 905 10.1007/s00198-011-1628-8 21625883 PMC3277698

[B36] Scheffer AC Schuurmans MJ van Dijk N van der Hooft T de Rooij SE Fear of falling: measurement strategy, prevalence, risk factors and consequences among older persons Age Ageing 2008 37 19 24 18194967 10.1093/ageing/afm169

[B37] Perera S Mody S Mody SH Woodman R Woodman RC Studenski S Studenski SA Meaningful change and responsiveness in common physical performance measures in older adults J Am Geriatric Soc 2006 54 743 749 10.1111/j.1532-5415.2006.00701.x 16696738

[B38] Hicks GE Shardell M Shardell M Alley DE Miller R Miller RR Bandinelli S Bandinelli S Guralnik J Guralnik J Lauretani F Simonsick EM Ferrucci L Ferrucci L Absolute strength and loss of strength as predictors of mobility decline in older adults: the InCHIANTI study J Gerontol A Biol Sci Med Sci 2012 67 66 73 21546582 10.1093/gerona/glr055 PMC3260485

[B39] Hays RD Woolley JM The concept of clinically meaningful difference in health-related quality-of-life research. How meaningful is it? Pharmacoeconomics 2000 18 419 423 10.2165/00019053-200018050-00001 11151395

[B40] Adachi JD Loannidis G Berger C Joseph L Papaioannou A Pickard L Papadimitropoulos EA Hopman W Poliquin S Prior JC Hanley DA Olszynski WP Anastassiades T Brown JP Murray T Jackson SA Tenenhouse A Canadian Multicentre Osteoporosis Study (CaMos) Research Group The influence of osteoporotic fractures on health-related quality of life in community-dwelling men and women across Canada Osteoporos Int 2001 12 903 908 10.1007/s001980170017 11804016

[B41] Mann CJ Observational research methods. Research design II: cohort, cross sectional, and case–control studies Emerg Med J 2003 20 54 60 10.1136/emj.20.1.54 12533370 PMC1726024

[B42] Gray CD Kinnear PR IBM SPSS Statistics 19 Made Simple 2012 Hove: Psychology Press

