# Supplementary material for: Postmenopausal women with osteopenia and a healed wrist fracture have reduced physical function and quality of life compared to a matched, healthy control group with no fracture
Source: BMC Womens Health. 2014 Aug 3;14:92. doi: 10.1186/1472-6874-14-92 (PMC4127073; doi:10.1186/1472-6874-14-92)
Supplement: Additional file 1 — Characteristics of patients with osteopenia and a healed wrist fracture (n = 18) and matched controls (n = 18). [file 1472-6874-14-92-S1.pdf]

Additional file 1: Characteristics of patients with osteopenia and a healed wrist fracture (n=18) and matched controls (n=18)

	Patients	Controls	Mean Difference (95% CI)	P Value
Age (years)	59.1	58.5	0.6 (-1.8 to 3.0)	0.613
Height (cm)	166.0	165.7	0.3 (-3.8 to 4.5)	0.869
Weight (kg)	67.5	66.6	0.9 (-2.9 to 4.7)	0.622
BMI (kg/m^2)	24.5	24.3	0.2 (-1.2 to 1.6)	0.741
Age of menopause (years)	50.5	49.8	0.7 (-0.2 to 1.6)	0.138
Years post menopause	8.7	8.7	0.0 (-1.9 to 1.7)	0.945
Wrist fracture:				
Right, n (%)	10			
Left, n (%)	8			
Hand dominance:				
Right, n (%)	17	16		0.579
Left, n (%)	1	2		
Body fat (kg)	24.5	24.0	0.5 (-3.9 to 4.9)	0.812
Body fat (%)	37.1	36.2	0.9 (-3.0 to 4.7)	0.638
Lean mass (kg)	40.9	40.5	0.4 (-2.5 to 3.3)	0.778
Lumbar spine (L_1-L_4) (T-score)*	-1.8	-0.6	-1.2 (-1.8 to -0.6)	<0.001
Hip total (T-score)	-1.5	-0.4	-1.1 (-1.4 to -0.7)	<0.001
Femur neck (T-score)	-1.6	-0.8	-0.8 (-1.2 to -0.5)	<0.001
Femur trochanter (T-score)	-1.4	-0.6	-0.8 (-1.3 to -0.4)	<0.001

Lumbar spine (L_1-L_4) (BMD g/cm^2)	0.948	1.114	-0.16 (-0.23 to -0.09)	<0.001
Hip total (BMD g/cm^2)	0.811	0.942	-0.13 (-0.18 to -0.07)	<0.001
Femur neck (BMD g/cm^2)	0.790	0.913	-0.12 (-0.17 to -0.07)	<0.001
Femur trochanter (BMD g/cm^2)	0.645	0.772	-0.12 (-0.18 to -0.07)	<0.001
PASE (0-315)	126.2	121.9	4.3 (-25.9 to 34.5)	0.770
Educational attainment :				
Higher degree > 3 (years), n (%)	9 (25)	9 (50)		1.0
Lower degree < 3 (years), n (%)	27 (75)	9 (50)		

Data are presented as mean,CI=Confidence Interval
**T-score=number of standard deviations below the mean of a healthy, young sex matched population [1]*
BMI=body mass index, BMD=bone mineral density, PASE=Physical Activity Scale for the Elderly
